# Criteria and non-criteria anti-phospholipid antibodies in the different clinical forms of antiphospholipid syndrome

**DOI:** 10.3389/fimmu.2025.1636171

**Published:** 2025-08-01

**Authors:** Oscar Cabrera-Marante, Daniel Pleguezuelo, Sara Garcinuño, Laura Naranjo, Raquel Díaz-Simón, Francisco Javier Gil-Etayo, Denis Zafra, Fernando Lozano-Morillo, Luis Morillas, Magdalena Abad, Olga Villar, Antonio Martínez-Salio, Tito Leoncio Lizarraga-Hurtado, Estela Paz-Artal, Antonio Serrano, Manuel Serrano

**Affiliations:** ^1^ Immunology Department, Hospital Universitario 12 de Octubre, Madrid, Spain; ^2^ Facultad HM de Ciencias de la Salud, Universidad Camilo José Cela, Villanueva de la Cañada, Madrid, Spain; ^3^ Instituto de Investigación Sanitaria HM Hospitales, Madrid, Spain; ^4^ Instituto de Investigación Sanitaria Hospital 12 de Octubre (imas12), Madrid, Spain; ^5^ Internal Medicine Department, Hospital Universitario 12 de Octubre, Madrid, Spain; ^6^ HLA and Molecular Biology Laboratory, Hospital Universitario de Samanca, Salamanca, Spain; ^7^ Hematology Department, Hospital Universitario 12 de Octubre, Madrid, Spain; ^8^ Rheumatology Department, Hospital Universitario 12 de Octubre, Madrid, Spain; ^9^ Gynecology Department, Hospital Universitario 12 de Octubre, Madrid, Spain; ^10^ Neurology Department, Hospital Universitario 12 de Octubre, Madrid, Spain; ^11^ Occupational Medicine, Hospital Universitario 12 de Octubre, Madrid, Spain

**Keywords:** anti-phospholipid antibodies, anti-phosphatidylserine/prothrombin, lupus anticoagulant, anti-phospholipid syndrome, thrombosis

## Abstract

**Introduction:**

Antiphospholipid syndrome (APS) is a systemic autoimmune disorder characterized by thrombotic symptoms (venous, arterial, or small vessels) and/or gestational morbidity in patients carrying antiphospholipid antibodies (aPLs). Criteria aPLs include anti-cardiolipin antibodies, anti-beta 2 glycoprotein I (aB2GPI) antibodies of the IgG or IgM isotypes, and lupus anticoagulant (LA). However, there are aPLs that are associated with APS events but are not included in the criteria (extra-criteria). The aim of this study is to evaluate the prevalence and association of criteria and extra-criteria aPLs with APS clinical events.

**Methods:**

A total of 838 patients with clinical manifestations of APS were studied. In total, 715 presented with vascular manifestations, and 130 presented with obstetric morbidity. We measured levels of criteria aPLs, and the extra-criteria aPLs determined were anti-phosphatidylserine/prothrombin (aPS/PT) of IgG and IgM isotypes and aB2GPI IgA.

**Results:**

Classic aPL, aPS/PT, and aB2GPI IgA positivity showed a significant and independent association with thrombosis (OR: 2.40, 2.36, and 2.53 respectively). IgA aB2GP1 was the only aPL significantly associated with the five types of thrombotic events (venous thrombosis, pulmonary embolism, stroke, acute myocardial infarction, and arterial thrombosis). Regarding obstetric APS, the most relevant antibodies were classic aPL of IgM isotype (OR: 36.04) and aPS/PT of both isotypes (OR: 4.4). The other aPL studied did not show association in multivariate analysis.

**Discussion:**

The degree of clinical association for each group of aPLs was different depending on the form of presentation (vascular or obstetric) and the presence or absence of autoimmune diseases. Moreover, a fair level of agreement between LA and aPS/PT positivity was found; therefore, aPS/PT should not be referred to as a surrogate marker of LA.

## 1 Introduction

In 1983, Graham Hughes described the antiphospholipid syndrome (APS), a multisystem autoimmune disorder characterized by thrombotic symptoms (venous, arterial, or small vessels) and/or gestational morbidity in patients carrying antiphospholipid antibodies (aPLs) (1).

Although APS was initially described in patients with systemic lupus erythematosus (SLE), nowadays, two clinical forms of APS are recognized (2): 1) SAD-APS (or PoliAu-APS), associated with other systemic autoimmune diseases, and 2) primary APS (P-APS), without any association with autoimmune diseases (3–5). Moreover, there is another entity, catastrophic APS (C-APS), a severe and rapidly progressive form with multiple thromboses and high mortality (6, 7).

There are no diagnostic criteria for APS. Classification criteria were established in the 8th International Congress of aPL (Sapporo, Japan, 1998) and expanded at the 11th International Congress held in Sydney in 2004 (Sydney’ Criteria). To classify a patient as having APS, it was necessary to meet at least one clinical criterion and one laboratory criterion. The clinical criterion is vascular thrombosis or gestational morbidity. The laboratory criterion is the presence of at least one of the agreed antiphospholipid antibodies: lupus anticoagulant (LA), anti-cardiolipin (aCL), or anti-beta 2 glycoprotein I (aB2GPI) of the IgG or IgM isotypes (8). In 2023, new criteria have been defined (ACR/EULAR criteria). Some clinical criteria were included, but there was a reduction in the laboratory criteria. The presence of aPLs of the IgM isotype has no value if it is not accompanied by aPLs of the IgG isotype (9).

Hughes and Khamashta defined “seronegative APS” as a group of patients with clinical criteria of APS, in which other pathologies have been excluded, but the aPL study is persistently negative. This situation may have several explanations, including the lack of sensitivity of the tests used to evaluate aPL antibodies (10), or the presence of aPL antibodies directed against another antigen or another isotype not included in the classification criteria (11) known as extra-criteria aPL or non-criteria aPL (12). Those with the strongest clinical association are anti-phosphatidylserine/prothrombin (aPS/PT) and anti-B2GP1 of the IgA isotype (IgA aB2GP1) (12, 13).

Several factors contribute to the exclusion of extra-criteria aPL from the classification criteria. For aPS/PT antibodies, there is a notably limited variety of diagnostic kits currently available, and for IgA aB2GP1, there is a lack of homogeneity between different kits, which causes contradictory results (14). In addition, many studies are based on cohorts comprising a small number of patients. It is frequently observed that these groups contain a disproportionately higher percentage of women and individuals with concomitant autoimmune disorders compared to that of the general population.

The functional test LA holds significant value because there is a subgroup of patients who are only positive for it, and it is one of the risk factors most closely associated with clinical events (15). It is suggested that this may be related with heterogeneous autoantibodies. However, the antibodies responsible for this coagulation disorder remain unknown (16). Some groups suggest that aPS/PT antibodies are closely related with LA and could serve as a potential surrogate marker, but this is a topic of considerable controversy, and further studies are needed to evaluate this hypothesis (17, 18).

The main goal of the study is to evaluate the prevalence of criteria and extra-criteria aPL as well as their degree of association with the presence of APS-related clinical events. Secondary objectives are to determine whether there are differences in each type of aPL in the degree of association with the different clinical events of APS and to assess the suitability of using aPS/PT antibodies as a surrogate marker of LA.

## 2 Materials and methods

### 2.1 Study design

We evaluated all 4,487 patients referred to the Immunology Department at Hospital 12 de Octubre in Madrid to assess the presence of antiphospholipid antibodies.

Samples were collected between 1 January 2017 to 31 December 2018. All patients must accomplish the following inclusion criteria: Patients over 18 years with a history of at least one previous clinical event included in the Sidney APS clinical criteria and evaluated for criteria and extra criteria aPL. Patients with an active oncologic or infectious disease, history of thrombophilia, coagulation defects, or prolonged immobility were excluded. All patients with aPLs were tested for antinuclear antibodies, anti-nucleosome and anti-cyclic citrullinated peptide.

A total of 838 patients were recruited and classified into two groups according to the clinical manifestations for the analysis: 715 with vascular manifestations and 130 with clinical criteria of obstetric APS according to the Sydney Criteria. **Supplementary Figure S1** describes the study’s patient distribution algorithm.

### 2.2 Control populations

The analysis was performed also with two different control groups, according to each clinical situation. For vascular APS, we recruited a general reference group of 296 healthy individuals with a comparable age range, as patients, and from the same geographic area. This group included 95 volunteers recruited from people over 50 years who underwent a preoperative study for minor conditions not related with any major disease (such as ophthalmic cataract surgery). The other 201 healthy persons were blood donors. None of the volunteers had history of vascular pathologies, 12 (4.1%) of them presented an autoimmune disease, but none of them were severe.

The control group for gestational morbidity analyses (N = 454) was composed of 59 women of fertile age from the reference group and a cohort of 395 women with normal gestation used to define the cut-off points for antiphospholipid antibodies in obstetric APS studies (19).

### 2.3 Laboratory determinations

All samples were tested for aCL and aB2GPI (isotypes IgG and IgM) using the addressable laser bead immunoassay (ALBIA) BioPlex 2200 (Bio-Rad Laboratories, Hercules, CA, USA). The cut-off was established at 18 U/ml according to international guidelines that recommend using p99 from the studied population (8).

IgA aB2GP1 and aPS/PT antibodies were quantified by enzyme-linked immunosorbent assay (ELISA) using the QUANTA Lite ELISA kit (INOVA Diagnostics Inc., San Diego, CA, USA). The p99 was 20 U/ml for IgA aB2GP1, 30 U/ml for aPS/PT IgG, and 40 U/ml for aPS/PT IgM antibodies. ELISA procedures were performed on a Triturus^®^ automatic analyzer (Diagnostics Grifols, Barcelona, Spain). Briefly, samples are diluted and incubated for 30 min. After that, the wells are washed three times. Later, the conjugated antibody is added and incubated for 30 min. Then, the wells are washed the same way, and substrate is added. After 30 min, the reaction is stopped, and the absorbance is read at 450 nm. This methodology ensures reproducibility and aligns with current best practice for aPS/PT antibody detection (20).

LA was measured in human citrated plasma according to the recommendations of the ISHT (21, 22) using, in all cases, two different tests: HemosIL dRVVT Screen and HemosIL dRVVT Confirm, and HemosIL Silica Clotting Time assays (Instrumentation Laboratory SpA, Milano, Italy). Before performing these tests, the patient’s condition was reviewed to avoid any associated infectious symptoms, and oral anticoagulation was discontinued.

Laboratory determinations were performed at a median of 224 (91–1,040) days of distance from the event.

### 2.4 Databases and statistical methods

Demographic and clinical data were obtained from the clinical records and stored in a work database. Immunological data, cardiovascular risk factors, presence of comorbidities, and previous therapies were stored in the database. Qualitative variables were described by absolute frequency and percentage. The relationship between cases and controls was studied using Pearson’s χ2 test or Fisher’s exact test where appropriate. Quantitative variables were described by median and interquartile range (IQR). The relationship between cases and controls was determined using the non-parametric Mann–Whitney U test.

To establish the association with the independent variables, binary logistic regression was performed in a univariate manner and quantified using the odds ratio (OR) along with its 95% confidence interval (CI). Multivariable analyses were performed using a logistic regression model and were accompanied by the area under the diagnostic performance curve or ROC (receiver operating characteristic) curve along with its 95% CI.

The degree of agreement between the presence of LA and aPS/PT was evaluated using Cohen’s kappa index (indicating the 95% confidence interval). To categorize the results of the kappa index, the scale proposed by Landis and Koch was used, adjusted to the five levels of the scale, depending on the intensity of the association. A kappa index of 0 to 0.20 was defined as slight, between 0.20 and 0.40 as fair, between 0.40 and 0.60 as moderate, between 0.6 and 0.8 as substantial, and above 0.8 as almost perfect (23).

The p-values lower than 0.05 were considered significant. Data analysis was performed using MedCalc 22.004 (MedCalc Software, Ostend, Belgium).

## 3 Results

### 3.1 Comorbidities in patients

The reference group had a median age of 56 years (IQR: 44–69) with a proportion of women of 53.4%. No significant differences were observed compared to patients (53 years, p = 0.185 and 54.5% of females, p = 0.732) (**Supplementary Table S1**).

Smoking and arterial hypertension were significantly more prevalent in patients than in controls (OR: 1.58, p = 0.006 and OR 1.45, p = 0.017 respectively). In the group of patients, 50 (6%) presented an additional systemic autoimmune disease (SAD). The most prevalent was SLE (2.3%), followed by systemic sclerosis (0.7%) and rheumatoid arthritis (0.6%). Seven patients (0.8%) presented other autoimmune diseases. No significant differences were observed in the prevalence of SAD when compared to controls (4.1%, p = 0.213; **Supplementary Table S1**).

The most commonly observed APS event was deep venous thrombosis (DVT) presented by 343 patients (40.9%). Pulmonary embolism (PE), stroke, acute myocardial infarction or arterial thrombosis were also represented with 23.9%, 20.4%, 6.6%, and 3.9% respectively.

Obstetric morbidity was observed in 130 patients (28.5% of the women and 15.5% of all the patients). Only 7 (5.4%) of these women presented also thrombotic events. Eleven percent of the patients presented more than one type of APS clinical manifestations. One male patient presented catastrophic APS.

### 3.2 aPL in controls and patients

The prevalence of criteria aPL in the reference group was 9.5%. Separating classic aPL and LA, the prevalences were 3.7% and 5.7%, respectively. The prevalence of extra-criteria aPL was 5.4% for IgA aB2GP1, 3.4% for aPS/PT IgM, and 2% for aPS/PT IgG (**Table 1**).

Considering all patients with clinical criteria, 187 (22.3%) were criteria aPL positive, 217 (25.9%) were extra-criteria aPL positive, 87 (10.3%) were positive for both types of aPL (**Supplementary Figure S1**), and 130 patients were positive only for extra-criteria aPL.

Consequently, 317 patients (37.8%) were positive for any aPL (criteria or extra-criteria). Of these 317 aPL-positive patients, 116 (37%) were APS-diagnosed patients who were evaluated for monitoring their disease, and 201 (63%) were identified for the first time as aPL carriers.

The presence of aPL in patients with clinical criteria was significantly higher than that of the controls (OR 2.75; 1.8–4.19). Considering also the extra-criteria aPL, the presence of aPL in the patient group was also higher (OR 2.60; 1.89–3.60).

**Figure 1:**
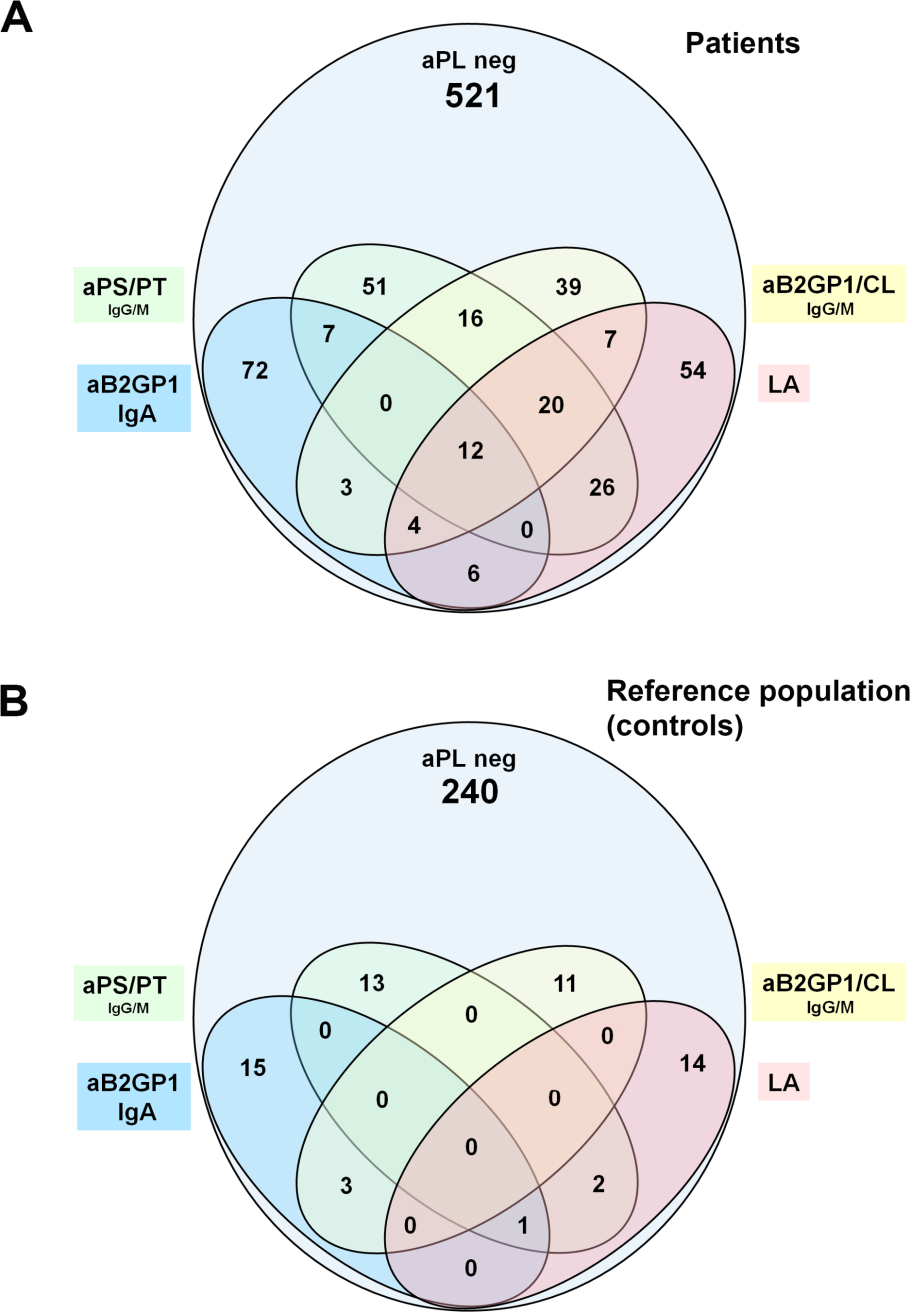
**(A)** Distribution of single and multiple aPL positivity in patients with APS clinical criteria. **(B)** Distribution of aPL in healthy reference population (controls).

The most prevalent aPL in patients was LA (15.4%). After this functional test, the most represented aPL were extra-criteria aPL: aB2GPI IgA (12.4%), aPS/PT IgM (11.1%), and aPS/PT IgG (8.1%). IgM isotype antibodies prevailed over IgG isotypes: aCL IgM (6.7%) vs. IgG (5.8%) and aB2GPI IgM (6.3%) vs. IgG (5.7%) (**Table 1**). The distribution of aPL positivity in the reference and patient groups are described in **Figures 1A, B**.

**Table 1:** Characteristics of patients with vascular APS clinical criteria versus controls from the general population.

Condition	Vascular APS criteria N = 715		General population N = 296		p-Value	OR	95% CI
	N/median	IQR/%	N/median	IQR/%			
Age (years)	57	45–70	56	44–69	0.220		
Sex (women)	334	(46.7%)	158	(53.4%)	0.054		
Dyslipidemia	211	(29.5%)	61	(20.6%)	0.004	1.61	1.17–2.23
Diabetes mellitus	76	(10.6%)	29	(9.8%)	0.693		
Smoking habit	208	(29.1%)	30	(10.1%)	<0.001	3.64	2.41–5.48
Arterial hypertension	243	(34%)	67	(22.6%)	<0.001	1.76	1.29–2.41
Obesity	29	(4.1%)	18	(6.1%)	0.164		
Additional autoimmune disease	37	(5.2%)	12	(4.1%)	0.450		
Antiphospholipid antibodies	162	(22.7%)	28	(9.5%)	<0.001	2.8	1.83–4.3
Lupus anticoagulant	113	(15.8%)	17	(5.7%)	<0.001	3.08	1.81–5.23
Classic aPL (aβ2GPI + aCL IgG/M)	86	(12%)	11	(3.7%)	<0.001	3.54	1.86–6.74
aβ2GPI IgA	96	(13.4%)	16	(5.4%)	<0.001	2.71	1.57–4.69
aPS/PT (IgG/M)	111	(15.5%)	16	(5.4%)	<0.001	3.22	1.87–5.54
APS events*							
Deep venous thrombosis	343	(48%)					
Pulmonary embolism	33	(4.6%)					
Arterial thrombosis	200	(28%)					
Stroke	171	(23.9%)					
Myocardial infarction	55	(7.7%)					
Obstetric morbidity*	7	(1%)					

IQR, interquartile range.

*Some patients have more than one event.

The most frequent isolated aPL positivity was IgA aB2GP1 with 72 (8.6%) followed by LA with 54 (6.4%) and aPS/PT with 51 (6.1%) (**Figure 1A**).

The number of patients with triple aPL positivity were 36 (4.3%) (**Supplementary Table S1**). A higher proportion of triple aPL positivity was observed in patients with autoimmune compared to patients without autoimmune diseases 14% versus 3.7% (p < 0.001).

One hundred thirty patients positive for extra-criteria aPL could not be detected using only standard criteria aPL studies. The distribution of serum levels of the different aPLs is described in **Supplementary Figure S2**.

### 3.3 Clinical association of the aPL with thrombotic events

Comparing vascular APS patients with the healthy population, all aPLs (criteria and extra-criteria) were significantly associated with higher rates of thrombi (**Table 1**). Patient distribution according to aPL positivity in patients with thrombosis is described in **Supplementary Figure S3A**.

Regarding cardiovascular risk factors, only smoking habits, dyslipidemia, and arterial hypertension were significantly associated with development of thrombotic events (**Table 1**).

For the first multivariate analysis, we considered the positivity of any classic aPL as a single variable (classic aPL) to increase the robustness of the study. We also unified the aPS/PT of both isotypes in a single variable (aPS/PT). The multivariate analysis included the factors that in the previous univariate analysis were associated with thrombotic events significantly or close to significance (p-value <0.1). Active smoking (OR: 3.31; 2.17–5.06) and high blood pressure (OR: 1.42; 1.01–1.99) were identified as independent variables significantly associated with thrombotic events.

Classic aPL and LA positivity showed a significant and independent association with thrombosis (OR: 2.40; 1.22–4.72) and (OR: 1.98;1.13–3.48), respectively.

The extra-criteria aPL were also shown to be independently associated with thrombotic events. The aPS/PT showed levels of association very similar to classic aPL (OR 2.36, 1.33–4.21), and the aB2GPI IgA showed the greatest level of association among the aPLs evaluated (OR: 2.53; 1.44–4.46) (**Table 2A**).

**Table 2:** Multivariate analyses of factors associated with vascular clinical criteria of antiphospholipid syndrome (APS).

A. Global vascular events multivariate												
Variable			Univariate				Multivariate					
			OR		95% CI		OR		95% CI		p-Value	
Sex (female)			0.77		0.58–1		0.86		0.65–1.15		0.324	
Active smoking			3.64		2.41–5.48		3.31		2.17–5.06		<0.001	
Arterial hypertension			1.76		1.29–2.41		1.42		1.01–1.99		0.046	
Dyslipidemia			1.61		1.17–2.23		1.24		0.87–1.77		0.237	
Lupus anticoagulant			3.08		1.81–5.23		1.98		1.13–3.48		0.017	
Classic aPL (aβ2GPI or aCL IgG/M)			3.54		1.86–6.74		2.40		1.22–4.72		0.011	
Anti β2GPI IgA			2.71		1.57–4.69		2.53		1.44–4.46		0.001	
Anti PS/PT (IgG or IgM)			3.22		1.87–5.54		2.36		1.33–4.21		0.004	
Area under the ROC curve (AUC)			0.703		(0.673–0.732)							

B. Independent event multivariate												
Variable	Venous thrombosis			Pulmonary embolism		Stroke		Myocardial infarction			Arterial Thrombosis*	
	OR	95% CI		OR	95% CI	OR	95% CI	OR		95% CI	OR	95% CI
Sex female	0.77	0.58–1		1.21	0.8–1.81	1.28	0.84–1.95	0.82		0.4–1.71		
Active smoking	3.64	2.41–5.48		4.32	2.59–7.2	3.01	1.75–5.18	8.15		3.79–17.49		
Arterial hypertension	1.76	1.29–2.41		1.77	1.12–2.8	1.29	0.79–2.09	1.63		0.76–3.53		
Dyslipidemia	1.61	1.17–2.23		1.52	0.95–2.42	1.28	0.78–2.09	3.60		1.69–7.67		
Lupus anticoagulant	3.08	1.81–5.23		2.56	1.28–5.13	1.47	0.68–3.18	1.94		0.65–5.77	2.40	0.74–7.75
Classic aPL aβ2GPI or aCL IgG/M	3.54	1.86–6.74		2.17	0.93–5.06	4.41	2.06–9.45	4.16		1.33–13.04	3.22	0.81–12.72
Anti β2GPI IgA	2.71	1.57–4.69		2.38	1.17–4.83	2.40	1.16–4.99	4.04		1.43–11.44	4.45	1.52–13.01
Anti PS/PT IgG or IgM	3.22	1.87–5.54		1.95	0.94–4.04	2.01	0.95–4.27	2.00		0.69–5.83	4.18	1.48–11.8
AUC	0.69	0.65–0.73		0.721	0.68–0.761	0.68	0.64–0.72	0.85		0.81–0.89	0.666	0.61–0.72

(A) Analysis of factors identified in the initial univariate analysis with a p-value < 0.1. (B) Analysis stratified by individual pathologies associated with APS. The only factors that remained as independent variables were smoking habit and the presence of IgA aB2GP1.

*Due to the low number of arterial thrombosis studied, only four variables could be analyzed, and the four types of aPLs were chosen. The rest of the factors were not analyzed. Multivariate variable aPL was associated with thrombosis. Shaded values did not obtain statistical significance.

### 3.4 Clinical association of aPL with different thrombosis manifestations

By analyzing separately each type of vascular APS event (**Table 2B**), we observed that in DVT, the four groups of antibodies, LA, classical aPL, IgA aB2GPI and aPS/PT were independent and significantly associated with an OR of 3.08, 3.54, 2.71, and 3.22, respectively. In pulmonary embolism, only LA (OR: 2.56) and IgA aB2GP1 (OR: 2.38) were identified as independent risk factors. Stroke and myocardial infarction were significantly associated with classic aPL (OR: 4.41 and 3.16) and IgA aB2GP1 (OR: 2.4 and 4.04). In arterial thrombosis, IgA aB2GP1 and aPS/PT were the aPLs that were significantly associated.

**Figure 2:**
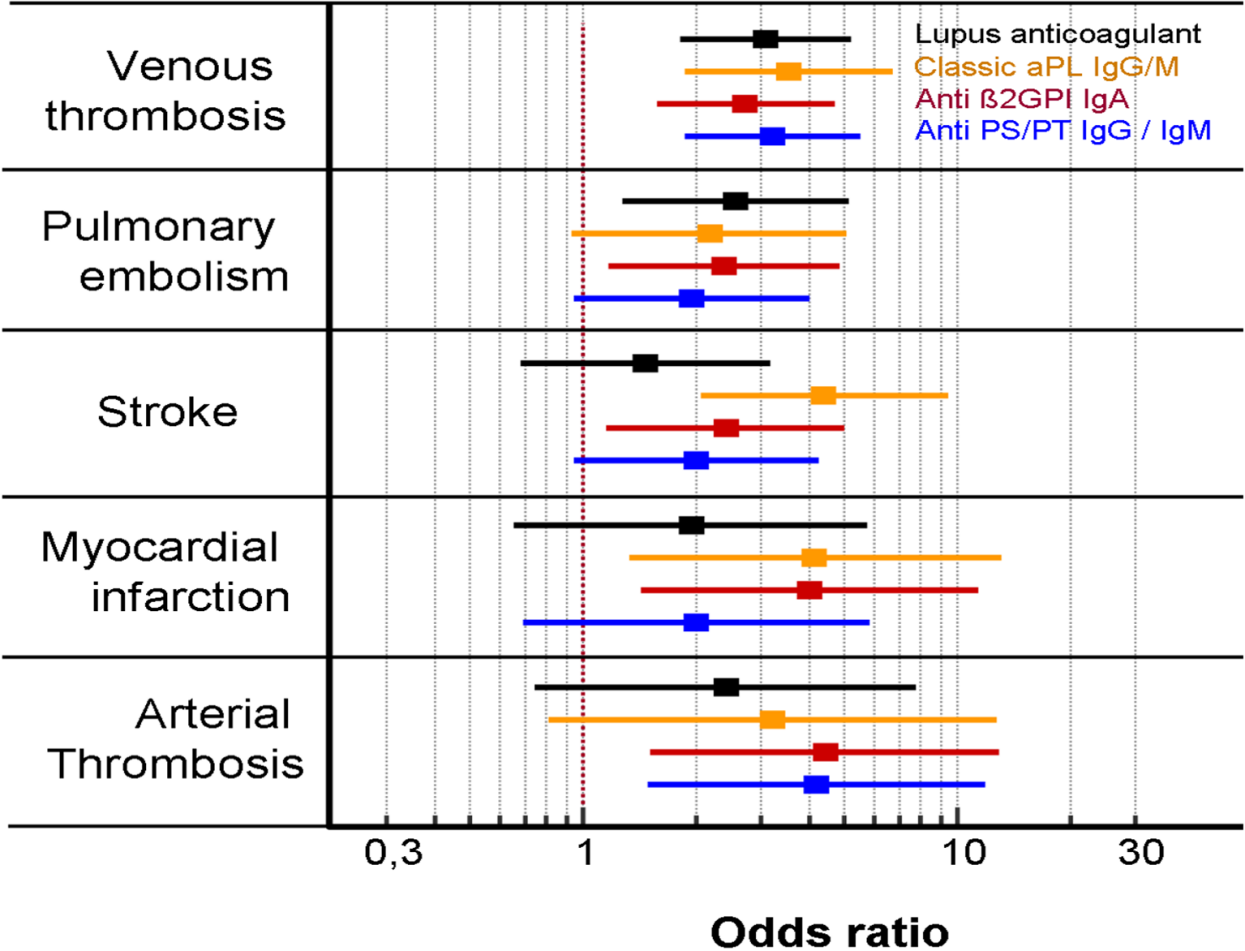
Association of aPL with each type of thrombotic events of the vascular antiphospholipid syndrome (multivariate analysis).

IgA aB2GP1 was the only aPL that was significantly associated to the five types of thrombotic events (**Figure 2**, **Table 2B**).

### 3.5 Clinical association of each aPL and pregnancy morbidity

According to obstetric events, of the 130 women with obstetric APS, 34 presented with ≥1 unexplained fetal death at or beyond the 10th week of gestation, 4 suffered pre-eclampsia, and 92 had ≥3 unexplained consecutive abortions. The 130 women with pregnancy morbidity were significantly older than the obstetric controls (37 vs. 32 years; p < 0.001). Of them, 40 (31%) were positive for at least one aPL: 11 for criteria aPL, 14 for extra-criteria aPL, and 15 for both. The prevalence of all aPLs was significantly higher in the patient group, especially for classic aPL (12.3% vs. 0.7%; OR: 21.1, p < 0.001).

In classic aPL, the IgM isotype was most prevalent (OR: 37.75; 4.79–298) showing a different profile from APS vascular patients. Moreover, there were no significant difference in the presence of LA in patients and controls. The most important extra-criteria aPLs were aPS/PT with an OR of 6.76 (3.36–13.57). Regarding cardiovascular risk factors, in the patient group, a higher proportion of dyslipidemia (OR 9.04; 1.73–47.15), arterial hypertension (OR: 4.57; 1.85–11.29), and SAD (OR: 6.10, 95% CI 2.61–15.3) was found (**Table 3**). Distribution of single and multiple aPL positivity in patients with obstetric morbidity is described in **Supplementary Figure S3B**.

**Table 3:** Characteristics, risk factors, and aPL prevalence in patients with obstetric morbidity versus a control group of pregnant women who had a normal pregnancy and delivery.

	Obstetric APS symptoms N = 130		Healthy women N = 454		p-Value	OR	95% CI
CONDITION	N/median	IQR/%	N/median	IQR/%			
Age (years)	37	33–40	32	27–36	<0.001		
Dyslipidemia	5	(3.8%)	2	(0.4%)	0.007	9.04	1.73–47.15
Diabetes mellitus	2	(1.5%)	4	(0.9%)	0.273		
Smoking habit	7	(5.4%)	37	(8.1%)	0.292		
Arterial hypertension	11	(8.5%)	9	(2%)	<0.001	4.57	1.85–11.29
Obesity	1	(0.8%)	10	(2.2%)	0.196		
Additional autoimmune disease	13	(10%)	8	(1.8%)	<0.001	6.19	2.51–15.3
Lupus anticoagulant*	17	(13.1%)	2	(3.4%)	0.0242	4.29	0.96–19.2
Classic aPL (aβ2GPI + aCL IgG/M)	16	(12.3%)	3	(0.7%)	<0.001	21.1	6.04–73.65
aβ2GPI IgM	10	(7.7%)	0	(0%)	<0.001	79.2	4.6–1361
aβ2GPI IgG	10	(7.7%)	2	(0.4%)	<0.001	18.83	4.07–87.11
aCL IgM	9	(6.9%)	1	(0.2%)	<0.001	33.69	4.23–269
aCL IgG	8	(6.2%)	1	(0.2%)	<0.001	29.7	3.68–240
Classic aPL IgM (IgM aB2GPI or aCL)	10	(7.7%)	1	(0.2%)	<0.001	37.75	4.79–298
Classic aPL IgG (IgG aB2GPI or aCL)	9	(6.9%)	2	(0.4%)	<0.001	16.81	3.58–78.83
aβ2GPI IgA	9	(6.9%)	6	(1.3%)	<0.001	5.55	1.94–15.91
aPS/PT (IgG/M)	23	(17.7%)	14	(3.1%)	<0.001	6.76	3.36–13.57
aPS/PT IgM	17	(13.1%)	7	(1.5%)	<0.001	9.61	3.89–23.73
aPS/PT IgG	12	(9.2%)	7	(1.5%)	<0.001	6.49	2.5–16.86
Clinical characteristics**							
Normal fetus dead (>10 weeks)	33	(25%)					
Premature births (<34 weeks)	6	(5%)					
Three or more abortions (<10 weeks)	95	(73%)					

IQR, interquartile range.

*Lupus anticoagulant was evaluated only in 58 healthy women.

**Some women suffered more than one process.

In the multivariate analysis (**Table 4A**), independent variables were age (OR 1.1; 1.07–1.15), classic aPL (OR 15.36; 3.7–63.84), and aPS/PT (OR 4.45; 1.96–10.11).

**Table 4:** Multivariate analysis of factors that were found to be associated with obstetric APS with a p-value <0.1 in the first univariate analysis.

	Univariate		Multivariate		
A. Risk factors	OR	95% CI	OR	95% CI	p-Value
Age (years)	1.13	1.09–1.17	1.11	1.07–1.15	<0.001
Dyslipidemia	9.04	1.73–47.15	4.00	0.53–30.48	0.181
Arterial hypertension	4.57	1.85–11.29	1.79	0.58–5.53	0.309
Additional autoimmune disease	6.19	2.51–15.3	1.98	0.58–6.84	0.278
Classical aPL (aB2GPI/aCL IgG/M)	21.1	6.04–73.65	15.36	3.7–63.84	<0.001
aβ2GPI IgA	5.55	1.94–15.91	3.23	0.85–12.25	0.084
aPS/PT (IgG or IgM)	6.76	3.36–13.57	4.45	1.96–10.11	<0.001
Area under the ROC curve (AUC)			0.758	0.721–0.792	

B. Risk factors	OR	95% CI	OR	95% CI	p-Value
Age (years)	1.13	1.09–1.17	1.11	1.07–1.15	<0.001
Dyslipidemia	9.04	(1.73–47.15)	4.00	0.52–30.52	0.181
Arterial hypertension	4.57	(1.85–11.29)	1.81	0.59–5.54	0.298
Additional autoimmune disease	6.19	(2.51–15.3)	1.96	0.57–6.81	0.287
Classic aPL IgM (aB2GPI or aCL)	37.75	4.79–298	36.04	3.56–65.23	0.002
Classic aPL IgG (aB2GPI or aCL)	16.81	3.58–78.83	6.06	0.91–40.32	0.063
aβ2GPI IgA	5.55	1.94–15.91	3.23	0.85–12.28	0.086
aPS/PT (IgG or IgM)	6.76	3.36–13.57	4.4	1.93–10.06	<0.001
Area under the ROC curve (AUC)			0.758	0.722–0.793	

A. Positivity for classic aPL (those included in the Sidney criteria) without a single variable. B. Classic aPL was divided into two variables: one collecting positives for classic aPL of the IgM isotype (IgM aB2GPI or aCL) and the other for those of the IgG isotype (IgG aB2GPI or aCL).Given the power of the IgM isotype in the univariate analysis (strongest risk factors), a second multivariate analysis was carried out in which classic aPL was divided into two variables: one collecting positives for classic aPL of the IgM isotype and the other for IgG isotype.

Due to the difference between the OR of the different isotypes in the univariate analysis (**Table 3**), we made a new multivariate analysis (see **Table 4B**), in which the classic aPL were divided into IgM and IgG isotypes.

The OR for classic aPL IgM was 36.04 (3.56–65.23), and for IgG, it remained slightly above the level of significance (p = 0.063). The AUC was 0.758 (95% CI 0.722–0.793). Between the first and second analysis, except for the aPL, the rest of the variables remained very similar.

### 3.6 aPL profiles depending on clinical presentation in primary APS vs. PoliAU APS

**Figure 3:**
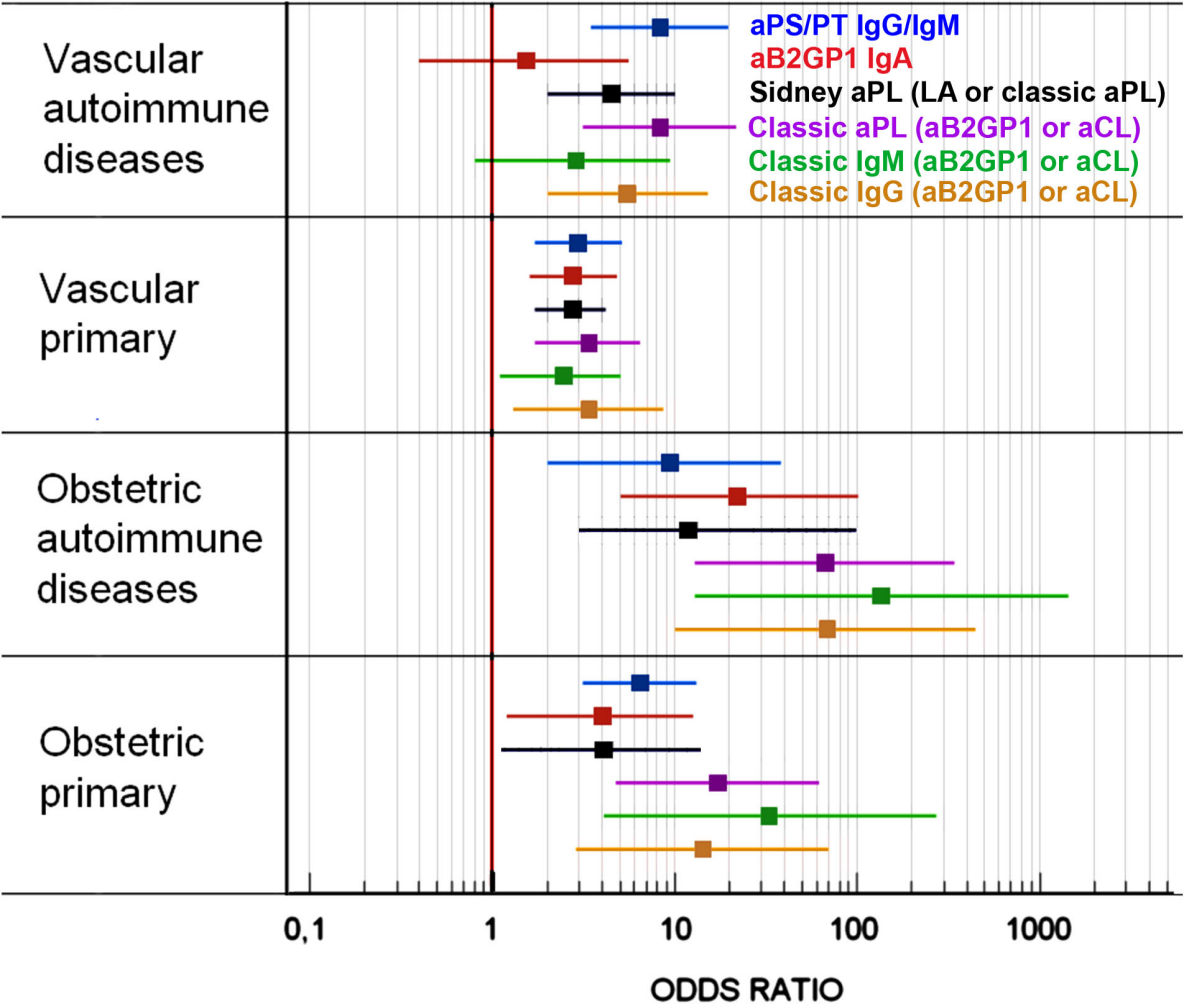
Strength of the association between the presence of the different aPLs and the disease in the most common clinical forms of APS.

All types of aPL were significantly associated with the primary vascular form and the obstetric form (both primary or associated with PoliAU). The degree of clinical association for each group of aPL was different depending on the form of presentation (vascular or obstetric) and the presence or absence of autoimmune diseases (**Table 5A**). Thrombotic APS with PoliAU did not show an association with IgA aB2GP1 (OR: 1.54; 0.43–5.57) nor with the classic aPL of the IgM isotype (OR: 2.87; 0.87–9.41) (**Table 5A**). However, classic aPL of the IgM isotype showed the strongest association in obstetric APS, both in the primary form (OR: 33.25; 4.11–268.64) and, notably, PoliAU form (OR: 136; 13–1,423). The association between the aPL with the clinical forms of the disease is described in **Figure 3**.

**Table 5:** A. Strength of the association between the presence of the different aPLs and disease in the more common clinical forms of APS.

	Primary form			*Autoimmune* disease associated		
A. aPL and symptoms profile						
Vascular symptoms (APS thrombotic form)						
Antibodies	OR	95% CI	p-Value	OR	95% CI	p-Value
aPS/PT (IgG/M)	2.96	1.71–5.11	<0.001	8.4	3.58–19.71	<0.001
aB2GPI I IgA	2.78	1.6–4.82	<0.001	1.54	0.43–5.57	0.208
Lupus anticoagulant	3.04	1.79–5.18	<0.001	3.83	1.47–9.97	0.006
Sidney aPL	2.72	1.77–4.18	<0.001	4.60	2.08–10.13	<0.001
Classic aPL (aB2GPI or aCL)	3.32	1.74–6.34	<0.001	8.33	3.18–21.81	<0.001
Classic IgM (aB2GPI or aCL)	2.46	1.19–5.08	0.012	2.87	0.87–9.41	0.066
Classic IgG (aB2GPI or aCL)	3.4	1.32–8.73	0.003	5.52	2.02–15.09	<0.001
Obstetric symptoms (gestational APS)						
Antibodies	OR	95% CI	p-Value	OR	95% CI	p-Value
aPS/PT (IgG/M)	6.48	3.16–13.28	<0.001	9.43	2.34–38.1	0.009
aB2GPI IgA	4.03	1.28–12.75	0.011	22.4	4.9–102.5	0.001
Lupus anticoagulant	3.20	0.69–14.8	0.137	17.5	2.89–106	0.002
Sidney aPL	4.01	1.14–14.05	<0.001	11.45	2.9–105.8	0.003
Classic aPL (aB2GPI or aCL)	17.18	4.76–61.97	<0.001	66.8	13–343	<0.001
Classic IgM (aB2GPI or aCL	33.25	4.11–268	<0.001	136	13–1,423	<0.001
Classic IgG (aB2GPI or aCL	14.38	2.95–70.19	<0.001	67.7	10.2–450	<0.001

B. Agreement lupus anticoagulant and anti-PS/PT						
Profile	Kappa	95% CI	p-Value	Kappa	95% CI	p-Value
Vascular or obstetric	0.338	0.249–0.426	0.024	0.343	0.058–0.629	0.015
Vascular symptoms (thrombosis)	0.323	0.233–0.423	<0.001	0.229	−0.084–0.563	0.183
Obstetric symptoms	0.425	0.198–0.655	<0.001	0.649	0.229–1	0.035

B. Strength of agreement between lupus anticoagulant markers and anti-phosphatidylserine/prothrombin antibodies (IgG/IgM) in the most common clinical forms of APS.

### 3.7 Correlation between aPS/PT and LA

132 patients tested positive for aPS/PT and 129 for LA. Seventy-four patients tested positive only for aPS/PT, 71 tested positive only for LA, and 58 patients (44%) were simultaneously positive for both tests. A fair level of agreement between LA and aPS/PT positivity was found (K: 0.342, CI: 0.258–0.426). When comparing the relationship between LA and each aPS/PT isotype separately, we found a kappa of 0.335 (CI: 0.246–0.424) for IgM and 0.233 (CI: 0.145–0.322) for IgG.

In patients with different clinical forms (**Table 5B**), we observed minimal differences in the kappa index when comparing agreement in primary APS (K: 0.338; CI: 0.249–0.426) versus PoliAu-APS (K: 0.343; CI: 0.058–0.629). Obstetric patients presented a better level of agreement (kappa: 0.470, CI: 0.263–0.677) compared to those with vascular symptoms (kappa: 0.322, 95% CI: 0.231–0.414). Notably, there is a substantial degree of agreement in the obstetric form associated with SAD, with a kappa of 0.649 (95% CI: 0.229–1) (**Table 5B**).

## 4 Discussion

In the present study, we demonstrate that the association of extra-criteria aPL with the Sydney APS clinical criteria is as relevant as criteria aPL. This occurs for both clinical forms of APS, vascular and obstetric. Furthermore, we found that the profile of aPL varies according to the clinical presentations of APS.

The proportion of patients with APS clinical criteria who were positive for at least one criteria aPL was 22.3%. If only new cases were considered, the prevalence is 16.7%, similar to 18% described in other works (24, 25). If patients with extra-criteria aPL were also considered, the prevalence of aPL increases to 37.47%. This finding reinforces the idea that the presence of aPL in the population with clinical manifestations of APS could be underestimated (26).

People with multiple autoimmune diseases are defined as PoliAu patients. It does not imply the existence of a disease subordinate to another main one (27). Approximately 1 in 20 patients (4.4%) with systemic autoimmune diseases (SAD) can be considered as PoliAu (28). In most studies about APS, the proportion of patients with additional autoimmune diseases is between 33% and 50% (9, 29). In this study, the size of the patient and control groups has allowed us to form subgroups of vascular APS (primary and poli-autoimmune) and obstetric APS (primary and poli-autoimmune), each with its own reference. We included, without bias, all samples from patients received in our laboratory, referred from all medical specialties due to the presence of a vascular or obstetric event. Thus, the proportion of patients with vascular form and additional autoimmunity (5.2%) is lower than that of other studies. This results are in line with previous studies in the same population (12, 30). Therefore, we propose that patients with PoliAu may be overrepresented in APS studies. This could lead to different proportions of autoimmune related APS between our studies and some of the cohorts reported in the literature (28).

The prevalence of autoantibodies (antinuclear, aPL, and antithyroid antibodies) in the general population becomes more prevalent as age increases without being associated with the presence of autoimmune diseases (31). They are considered to be scavenger antibodies that clean up apoptotic cells generated by tissue damage associated with the senescence process (32). To determine the real prevalence and minimize the influence of age as a variable of vascular pathology, we used a reference group with a similar age range as the patients.

There is a great heterogeneity in the results published in the scientific literature regarding the relevance of different aPLs especially for non-criteria aPLs. Among the factors that contribute to heterogeneity, the lack of harmonization between immunoassays stands out (10, 14).

Moreover, these discrepancies could also be due to imbalances in the composition of the populations used: populations enriched in PoliAu patients, which are more complex and exhibit clinical characteristics and specific biomarker (33). For this reason, we compare different subgroups particularly focusing on the differences between primary forms and those associated with other autoimmune conditions.

Classically, LA is considered the aPL with the greatest independent risk association in APS, especially in patients with vascular manifestations (34, 35). In this work, we observed that all IgG isotype antibodies, as well as the aPS/PT IgM, showed association with APS symptoms higher than that obtained by LA. Furthermore, we confirmed that the antibodies included in the Sydney criteria of the IgM isotype present a lower correlation with the clinical manifestations of vascular APS than those of the IgG isotype (17, 36–39). However, they are extremely important in obstetric APS.

In the primary vascular APS, all the tested aPLs were associated with clinical events, particularly IgA aB2GPI, as we described previously (40). In thrombotic PoliAu-APS patients, the classic aPL of the IgG isotype and aPS/PT were the most important aPLs, and no significant association was found with aPL of the IgM and IgA isotypes. This absence of association may be explained by the dominant role of molecular mimicry in primary APS proposed by other authors. This contrasts with the complex autoimmune milieu and regulatory mechanisms present in secondary APS, which may mask or override the pathogenic effects of IgA anti-β2GPI (41, 42).

IgA aB2GP1 is the only aPL that is independently associated with all APS vascular clinical events, especially those related with arterial thrombosis, such as myocardial infarction, stroke, and arterial thrombosis, as previously described (12, 43, 44). However, the association of IgA aB2GP1 with vascular events in patients with additional autoimmune diseases is not significant. This finding is explainable since patients with polyautoimmunity present different biomarker profiles than those with monoautoimmunity (33). This fact may explain why, in many studies with an overrepresentation of patients with autoimmune diseases, authors often do not find a strong association between this aPL and clinical symptoms.

The current 2023 ACR/EULAR classification criteria are perfectly adapted to the vascular APS form but are insufficient for obstetric APS (primary and PoliAu) and primary vascular APS. The possibility of establishing criteria adapted to the forms of the disease should be strongly considered, as is done in catastrophic APS.

In obstetric APS patients, the characteristics and degree of association of the different factors and aPL were different from vascular APS. When comparing women with obstetric morbidity with the controls, the influence of other demographic variables and comorbidities seems to be less relevant. The apparently contradictory result obtained for smoking as a protective factor could probably be explained by the indication of suppression of tobacco consumption in patients with obstetric morbidity. In the multivariate analysis, in addition to aPL, only age persists as a risk factor, a variable usually associated with lower live birth rates (45). In obstetric APS, both primary and autoimmunity associated, classic aPL of the IgM isotype were the most strongly associated aPL with the clinical manifestations. Therefore, taking into account the new classification criteria complicates the classification of these patients as APS.

aPS/PT demonstrated a level of clinical association independent of the rest of the variables, with an OR for obstetric morbidity of 4. This result is similar to previously published works in which the presence has been associated with aPS/PT with spontaneous and recurrent abortions, complex complications of pregnancy or prematurity (13, 46–50). This aPL could have a relevant added value in the prognosis of these patients. Moreover, the number of positive patients for this aPL in healthy control remains under 5.5% in both control groups. The testing of aPS/PT may be relevant for risk stratification, even in the presence of other antibodies, as it is included in the Global Antiphospholipid Syndrome Score (GAPSS) (51). Moreover, more studies are needed in patients with triple positive.

Previously, aPS/PT has been described as a surrogate marker for LA (17, 18, 52). In our study there exists a correlation, but it is minimal except for patients with obstetric form associated with autoimmunity where moderate agreement is observed. Both markers had a significant association with the clinical manifestations of APS, but independently. The number of patients who had both markers was much smaller than those who had each of the markers separately. The aPS/PT marker is acquiring clinical relevance as an independent aPL associated with APS (47, 53). Many specialized centers already use it, although it is not included in the classification criteria (15, 54, 55).

The proportion of women with obstetric and vascular manifestations is very small, which suggests that obstetric APS is a clearly different transient form of the disease. Since it only manifests in women of gestational age, prophylactic treatments would not be indicated outside of these stages.

This study has several limitations: it has been designed as a cross-sectional study in which only one center was included. Due to the nature of the study and ethical conditions, it has not been possible to access clinical and analytical data on the evolution of the patients, and aPL has only been assessed on one occasion. However, the laboratory’s reference data for classic aPL and IgA aB2GP1 show that 95% of those positive continue to be positive 3–6 months after the first analysis (25).

The number of obstetric controls for whom LA could be determined was very small, and it was not enough to incorporate it into a multivariate analysis. Moreover, the number of patients with events and other autoimmune diseases is not very high. Therefore, the conclusions should be contrasted in future studies by expanding this cohort. Also, only one center was included. We have not used the ACR/EULAR classification criteria because the study was launched long before these criteria were published. Although the new clinical criteria represent a significant advance and the laboratory criteria are very well adapted for APS associated with autoimmune diseases, they are insufficient to recruit patients with primary APS, which, as we describe, is the most common form.

## 5 Conclusions

The degree of association of an aPL with APS pathology varies depending on the clinical forms of the disease, especially whether or not it is associated with other autoimmune processes.

The determination of IgA aB2GP1 and aPS/PT antibodies of the IgG and IgM isotypes allows the identification of patients at high risk for arterial or venous thrombosis. For this reason, its inclusion in the aPL detection profiles in patients with APS symptoms would help in the management of patients who are currently considered to have “seronegative antiphospholipid syndrome.” Although aPS/PT should not be used to replace LA, the evaluation of these antibodies might be a valuable diagnostic tool in patients with gestational morbidity when classic aPL or LA is negative or inconsistently positive.

The clinical value of the presence of classic aPL of the IgM isotype must be recovered, at least in the obstetric form of the disease.

## Data Availability

The raw data supporting the conclusions of this article will be made available by the authors, without undue reservation.
