# Supplementary material for: Criteria and non-criteria anti-phospholipid antibodies in the different clinical forms of antiphospholipid syndrome
Source: Front Immunol. 2025 Aug 1;16:1636171. doi: 10.3389/fimmu.2025.1636171 (PMC12354381; doi:10.3389/fimmu.2025.1636171)
Supplement: Supplementary file 1 [file DataSheet1.docx]

Supplementary Material

# Supplementary Figures


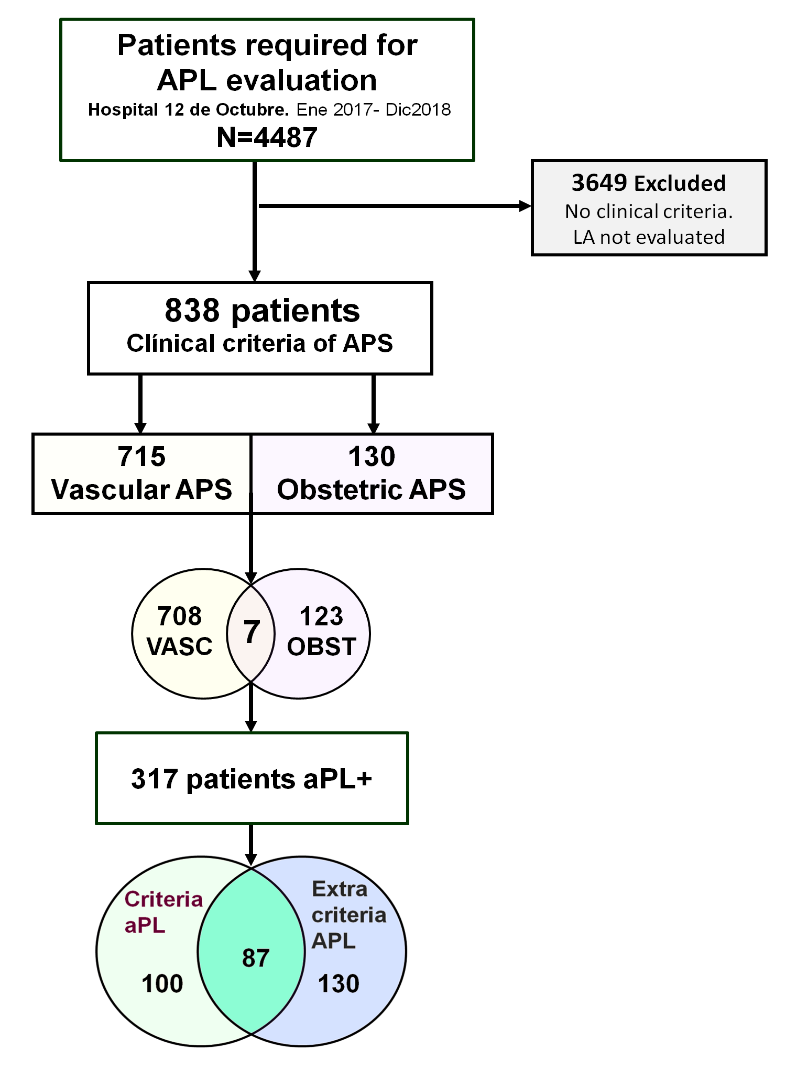


**Supplementary Figure S1**. Algorithm and distribution of the population with APS clinical criteria.


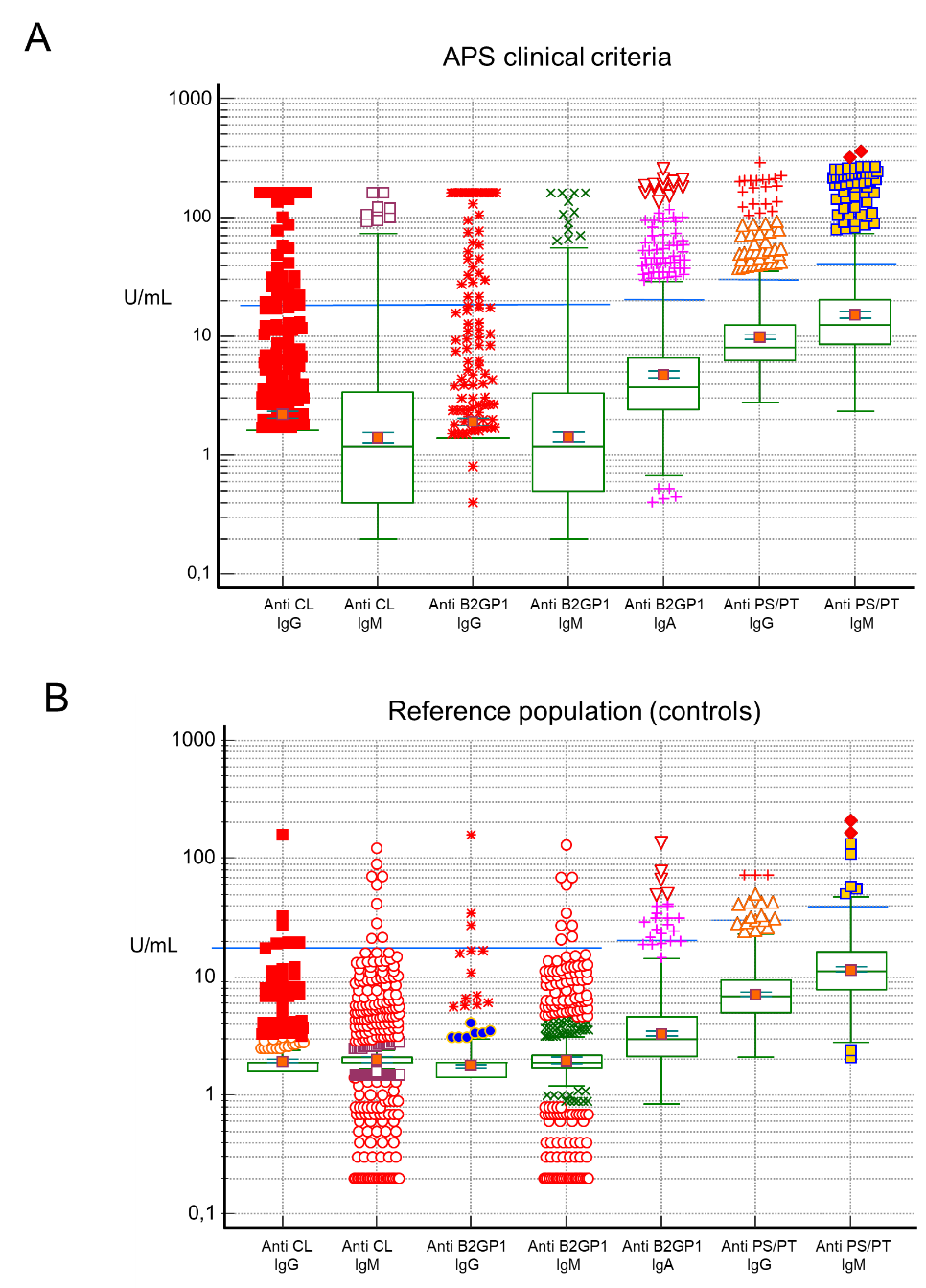


**Supplementary figure S2**. A Levels of aPL in patients with clinical criteria of APS. B. Levels in the reference population of 296 healthy people. The horizontal blue lines indicate the cutoff points for each antibody.


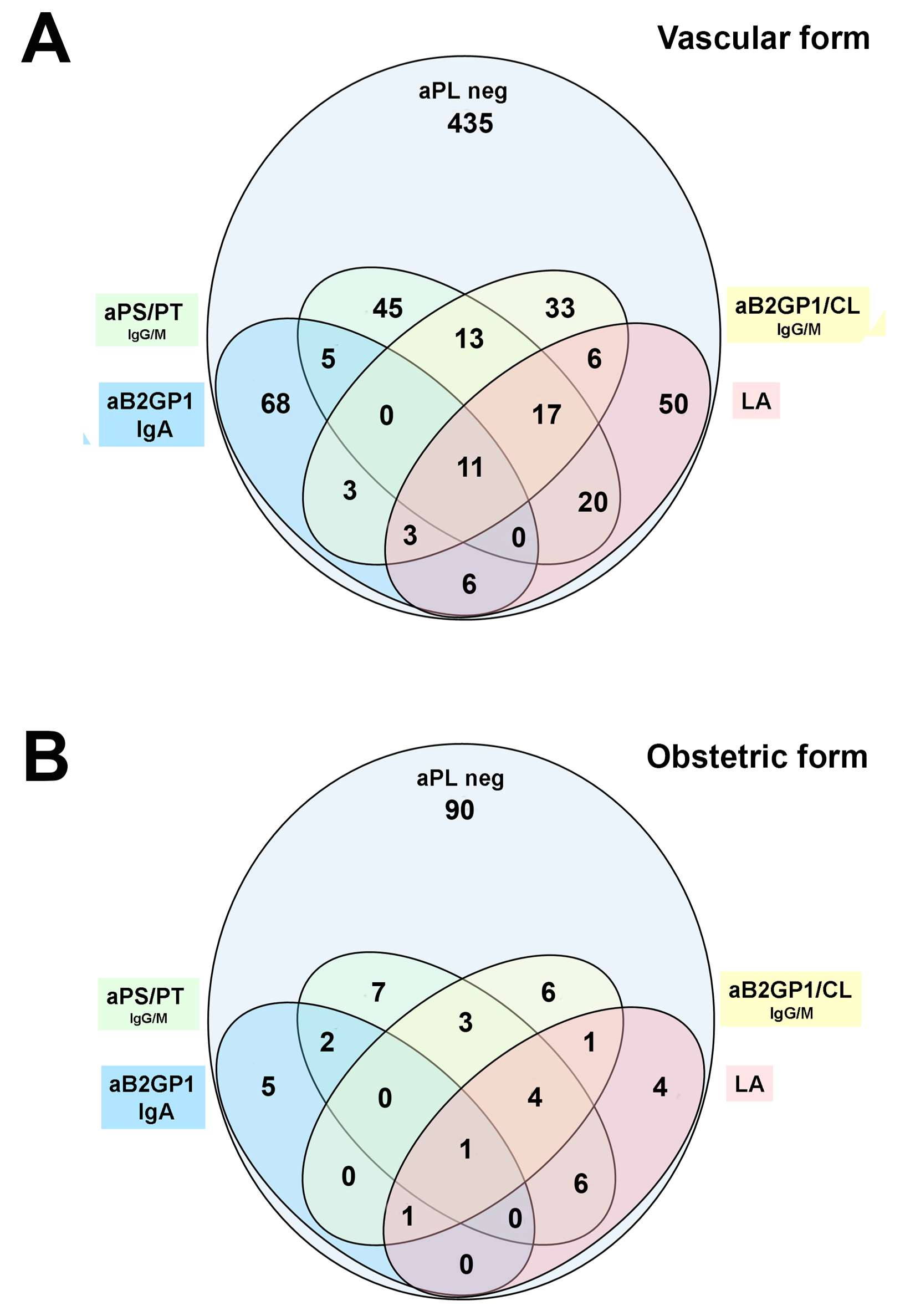


**Supplementary Figure S3** Venn diagram describing single and multiple aPL positivity in vascular and obstetric patients.

# Supplementary Tables

Supplementary Table S1: Demographic characteristics, cardiovascular risk factors and aPL prevalence in general population and in the group of patients with APS clinical criteria. IQR: Interquartile range

|  | General population  N=296 | | APS Symptoms  N=838 | | p | OR | 95% CI |
| --- | --- | --- | --- | --- | --- | --- | --- |
| CONDITION | N/median | IQR / % | N/median | IQR / % |  |  |  |
| Age (years) | 56 | 44-69 | 53 | 39-69 | 0.185 |  |  |
| Sex (women) | 158 | 53.4% | 457 | 54.5% | 0.732 |  |  |
| Dyslipidemia | 61 | 20.6% | 214 | 25.5% | 0.089 |  |  |
| Diabetes mellitus | 29 | 8.8% | 78 | 9.3% | 0.804 |  |  |
| Smoking habit | 57 | 19.6% | 229 | 27.3% | 0.006 | 1.58 | 1.14-2.19 |
| Arterial hypertension | 67 | 22.6% | 250 | 29.8% | 0.017 | 1.45 | 1.07-1.98 |
| Obesity | 18 | 6.08% | 29 | 3.5% | 0.076 |  |  |
| Additional systemic autoimmune disease | 12 | (4.1%) | 50 | (6%) | 0.213 |  |  |
| Systemic lupus erythematosus | 0 | (0%) | 19 | (2.3%) | 0.003 |  |  |
| Rheumatoid arthritis | 1 | (0.3%) | 5 | (0.6%) | 0.346 |  |  |
| Systemic sclerosis | 1 | (0.3%) | 6 | (0.7%) | 0.298 |  |  |
| Sjögren's syndrome | 0 | (0%) | 4 | (0.5%) | 0.298 |  |  |
| Psoriasis | 3 | (1%) | 3 | (0.4%) | 0.144 |  |  |
| Inflammatory bowel disease | 1 | (0.3%) | 6 | (0.7%) | 0.298 |  |  |
| Other autoimmune diseases | 6 | (2%) | 7 | (0.8%) | 0.098 |  |  |
| Sidney aPL | 28 | (9.5%) | 187 | (22.3%) | <0.001 | 2.75 | 1.8-4.19 |
| Lupus anticoagulant | 17 | (5.7%) | 129 | (15.4%) | <0.001 | 2.99 | 1.77-5.04 |
| Classic aPL ( aβ2GPI+aCL IgG/M) | 11 | (3.7%) | 101 | (12.1%) | <0.001 | 3.55 | 1.88-6.71 |
| aβ2GPI IgM | 8 | (2.7%) | 53 | (6.3%) | 0.018 | 2.43 | 1.14-5.17 |
| aβ2GPI IgG | 2 | (0.7%) | 48 | (5.7%) | <0.001 | 8.93 | 2.16-36.98 |
| aCL IgM | 9 | (3%) | 56 | (6.7%) | 0.021 | 2.28 | 1.12-4.68 |
| aCL IgG | 5 | (1.7%) | 49 | (5.8%) | 0.001 | 3.61 | 1.43-9.16 |
| Triple positive | 1 | (0.3%) | 36 | (4.3%) | <0.001 | 13.24 | 1.81-97.02 |
| Non criteria aPL | 31 | (10.5%) | 217 | (25.9%) | <0.001 | 2.99 | 2-4.47 |
| aβ2GPI IgA | 16 | (5.4%) | 104 | (12.4%) | <0.001 | 2.48 | 1.44-4.27 |
| aPS/PT (IgG/M) | 16 | (5.4%) | 132 | (15.8%) | <0.001 | 3.27 | 1.91-5.6 |
| aPS/PT IgM | 10 | (3.4%) | 93 | (11.1%) | <0.001 | 3.57 | 1.83-6.95 |
| aPS/PT IgG | 6 | (2%) | 68 | (8.1%) | <0.001 | 4.27 | 1.83-9.94 |
| Any aPL (criteria or extra-criteria) | 56 | (18.9%) | 317 | (37.8%) | <0.001 | 2.60 | 1.89-3-60 |
|  |  |  |  |  |  |  |  |
